# Denaturation of the SARS-CoV-2 spike protein under non-thermal microwave radiation

**DOI:** 10.1038/s41598-021-02753-7

**Published:** 2021-12-03

**Authors:** Pooya Afaghi, Michael Anthony Lapolla, Khashayar Ghandi

**Affiliations:** Department of Chemistry, University of Gulph, 50 Stone Road E, Guelph, ON N1G 2W1 Canada

**Keywords:** Microbiology, Chemical biology

## Abstract

SARS-CoV-2, the virus that causes COVID-19, is still a widespread threat to society. The spike protein of this virus facilitates viral entry into the host cell. Here, the denaturation of the S1 subunit of this spike protein by 2.45 GHz electromagnetic radiation was studied quantitatively. The study only pertains to the pure electromagnetic effects by eliminating the bulk heating effect of the microwave radiation in an innovative setup that is capable of controlling the temperature of the sample at any desired intensity of the electromagnetic field. This study was performed at the internal human body temperature, 37 °C, for a relatively short amount of time under a high-power electromagnetic field. The results showed that irradiating the protein with a 700 W, 2.45 GHz electromagnetic field for 2 min can denature the protein to around 95%. In comparison, this is comparable to thermal denaturation at 75 °C for 40 min. Electromagnetic denaturation of the proteins of the virus may open doors to potential therapeutic or sanitation applications.

## Main

Conceivably, some of the most innovative research related to the COVID-19 pandemic involves the characterization of the spike (S) protein of SARS-CoV-2 and its role during the first stages of infection. From here, a multitude of proposed methods were established surrounding this troublesome peplomer to interrupt its interaction with host cells, for instance, recombinant protein administration, drug applications, and vaccine intervention^1–7^. Vaccination against SARS-CoV-2 is the most substantial preventative measure against this virus that we presently have. Yet, in the case of such vaccine-elicited immunity, only limited effectiveness is attained as emergent variants may escape vaccine derived antibodies^8^. Perpetual development into “booster shots” as an updated version of the vaccine to compete with “updated” versions of the virus appears to be the proposed method to handle this situation, however, it is still unclear how long this cycle will persist. Additionally, infection can still occur despite vaccination, perhaps even more so when considering mutant variants^8–10^. Can we induce herd immunity rapidly enough through vaccination *en masse*, and how will we overcome the associated challenges^11^?

Until a more systematic answer is defined, novel methods for dealing with persisting contagions and rendering viruses non-functional should be explored, especially techniques with the ability to disable virions indiscriminate of variant. Such methods could also be used to disinfect surfaces contaminated with the virus. Here, we investigate the degree of denaturation (DoD) of the S1 subunit of the SARS-CoV-2 spike protein in solution under microwave radiation at body temperature, 37 °C. Due to the nature of electric field effects on proteins, this method should also be effective in denaturing current variants of SARS-CoV-2. An ultraviolet (UV) spectroscopy analysis technique that has been previously established was used to measure the degree of denaturation^12^. To that end, the thermal (heated in a water bath) denaturation of the S1 subunit was performed. This was used as a reference for comparison to the effect of electromagnetic field, and to provide quantitative information on the impact of temperature on spike protein.

## Experimental

**Figure 1:**
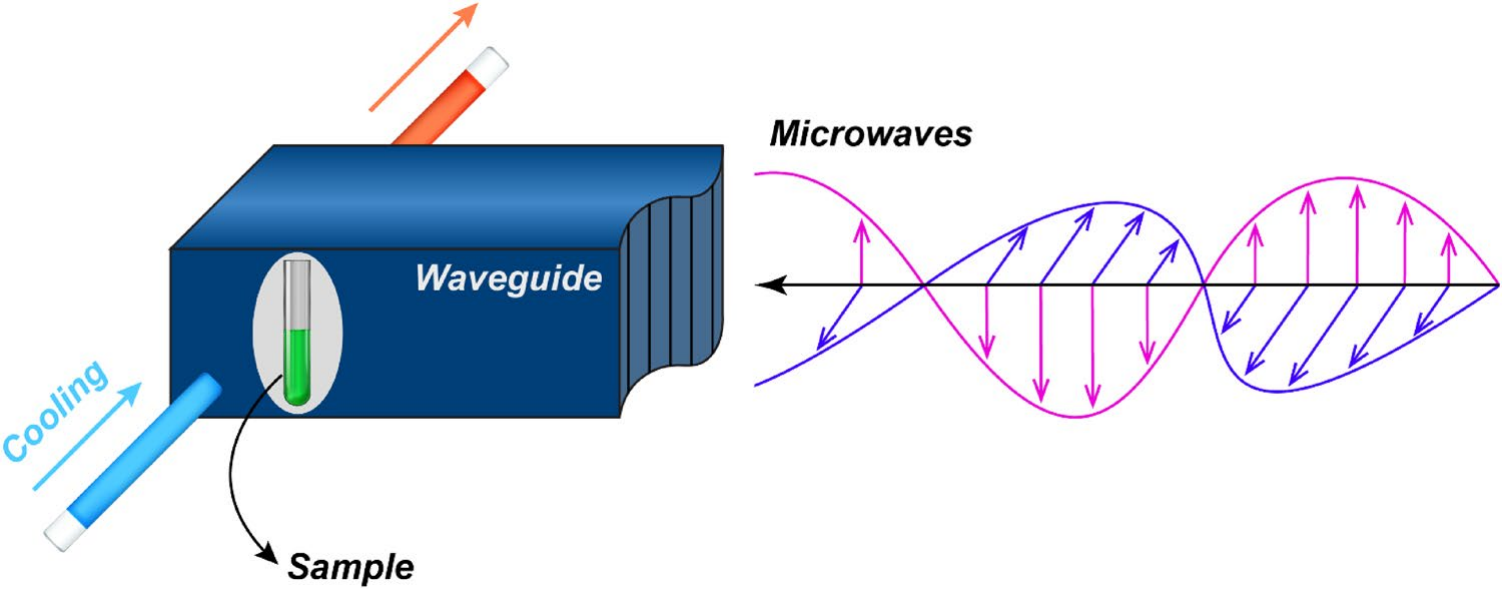
The sample is placed inside a microwave waveguide and connected to an efficient cooling system.

The thermal denaturation of SARS-CoV-2 S1 subunit was tested using a novel microwave system. Figure 1 shows a schematic of the setup where the sample is placed inside a microwave waveguide and connected to an efficient cooling system.

For all the protein experiments, the power of the microwave source was set to 700 W and the total microwave exposure time was 10 min. The temperature of the sample was kept at 37 °C with microwave exposure. The UV spectrum of the sample was obtained immediately after microwave exposure. Microwave irradiation and UV–visible techniques were replicated for the proteins subject to 2, 5, and 10 min of exposure time.

**Figure 2:**
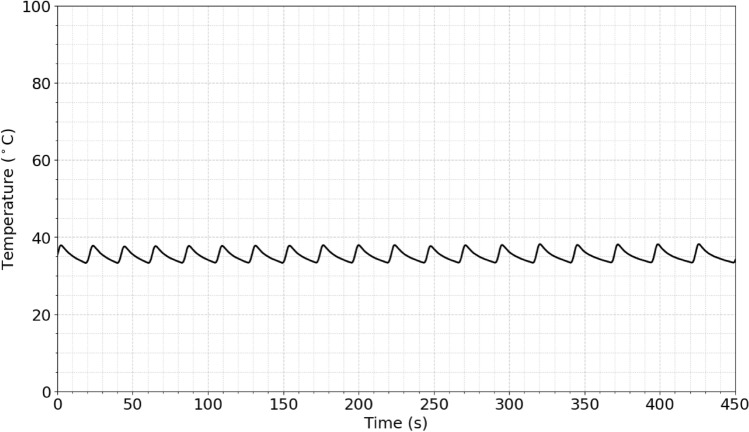
Stability of the temperature of the sample under high-power microwave radiation.

The temperature of the sample was measured in real-time and in situ by using a fast and accurate optical thermometer placed inside the sample solution. The sample chamber was a very small quartz tube (~ 3 × 20 mm) enclosed inside a secondary chamber where a coolant was circulated. Since the surface to volume ration was very high, the coolant was in a very good thermal contact with the sample. By automatic adjustments of microwave pulses and the use of coolant and special design described above, the average temperature of the sample was maintained at the desired level. Furthermore, the high contact surface of the fiber optic temperature probe (that was inserted in the sample) with the solution ensured the accurate temperature monitoring. Figure 2 shows the stability of the temperature measured in real-time using the sensitive optical fiber thermometer. This will prevent the temperature from rising above a certain threshold and enables us to eliminate the thermal effects of microwaves.

## Results and discussion

Proteins, in general, have amino acid residues such as tyrosine (Tyr), phenylalanine (Phe) and tryptophan (Trp). The aromatic chains of these residues have strong UV absorption in the range of 240–300 nm^13^. During the denaturation of a protein, as the protein unfolds, some of these residues are exposed to the solvent and are perturbed. This will cause absorption of higher energy photons and shifting of the absorption spectrum to the left. However, the portion of the UV spectrum that shifts to the left is small and difficult to quantify using conventional methods of peak height measurement, hence, the novel UV analysis technique was assigned to the task^12^.

In this study, first the temperature sensitivity of the protein to thermal denaturation was obtained at three different incubation temperatures and as a function of time. To our knowledge this is the first such kinetics studies. It was then compared with microwave radiation at body temperature. For instance, at 75 °C it took about 40 min for the protein to be almost fully denatured. However, one must consider that this study only pertains to the heating effects, pure electromagnetic effects and localized microscopic microwave heating effects. While there is a lack of extracellular species in these experiments that are normally present in the biological species, this work provides important initial steps to understanding such interactions between microwave radiation and the viral spike protein. In-vivo experiments that are indeed consistent with our results will be the topic for the future publications.

Temperature sensitivity of the SARS-CoV-2 spike protein has been also documented in previous literature in multiple molecular dynamic and in-vitro studies^14–16^, indicating significant inactivation of the virus at 56 °C^16^. Martí and colleagues report changes to the global S1-protein structure, and a substantial rearrangement of both the N-terminal domain (NTD) and receptor binding domain (RBD) as temperatures increased14. Spectral changes found here are consistent with the results found in above studies^14,15^.

The consistency of our spectroscopic results here (protein conformation changes with temperature) with the computational results in references 14 and 15, and with our previous works on other proteins^12^, suggests that the spectroscopic method is a reasonable indicator of the degree of denaturation. Therefore, the spectroscopic method was used to evaluate protein alterations under thermal or microwave exposure. Since the RBD is a crucial component in the spike protein for binding with the human ACE2 receptor, an increase in the degree of denaturation with temperature implies a substantial loss of binding affinity to human cells.

**Figure 3:**
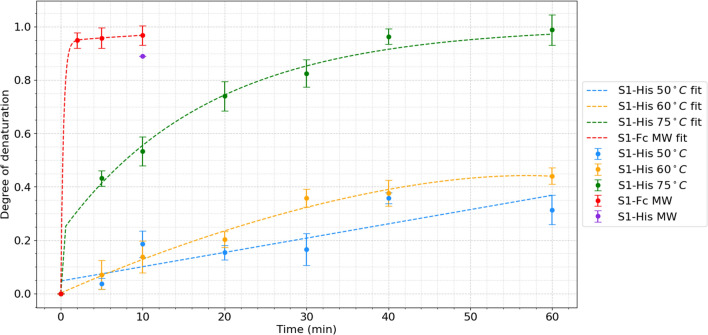
Degree of denaturation due to thermal heating of the S1 subunits compared to microwave radiation (37 °C) for each average temperature and time variable.

The gradual denaturation of the protein with incubation time and microwave exposure is displayed in Fig. 3.

**Figure 4:**
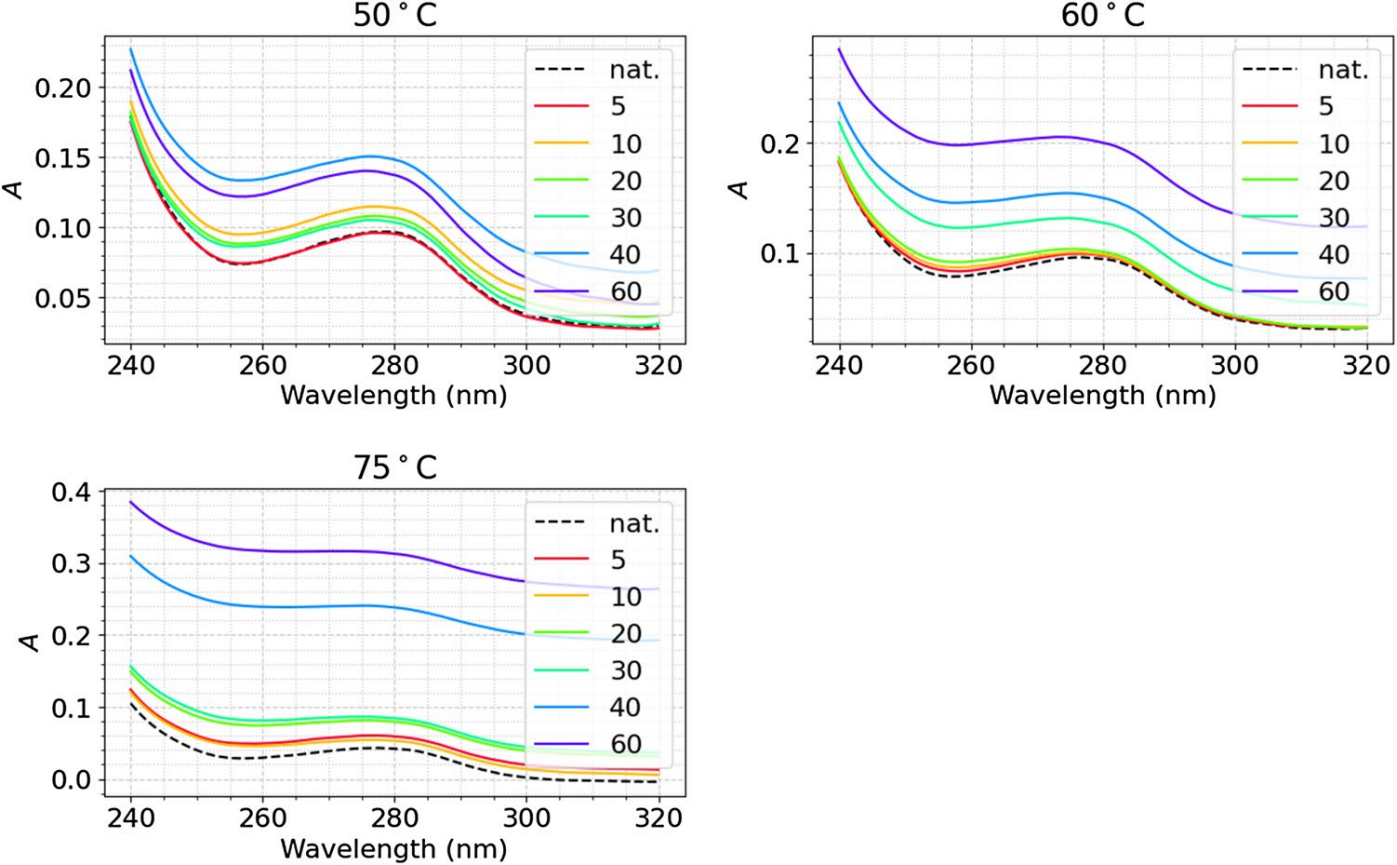
Absorption spectra of the SARS-CoV-2 spike protein S1-His recombinant subunit at average temperatures, 50, 60, and 70 °C at varying incubation times. Absorption maxima can be seen at ~ 277 nm for each.

The new data analysis technique used in this study will be described by using the thermal denaturation of the S1-His protein at 50 °C as an example. The raw curves of each temperature/time after smoothing are presented in Fig. 4. The peaks display broad and small shifts.

**Figure 5:**
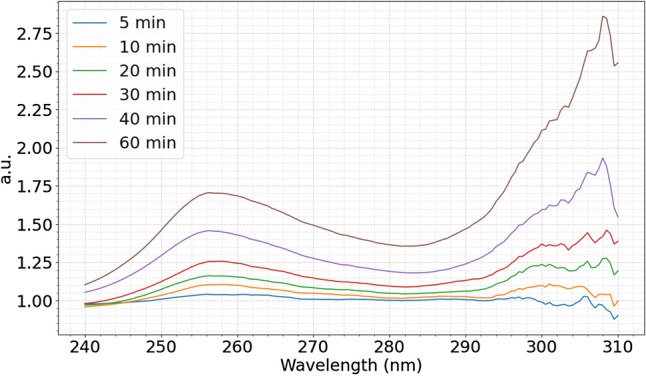
UV spectra shifts of the S1 protein at 50 °C at varying incubation times. Main peak centered at ~ 256.5 nm.

Using the 50 °C spectra of the S1-His protein, the main peak can be seen centered at around 277 nm. To analyze the denaturation, a mathematical analysis previously defined by the authors is used to yield the data displayed in Fig. 5^12^.

The mathematical model quantifies the portion of the shift of the peak centered at 277–256.5 nm due to the perturbation of amino acid residues when they are exposed to water as the protein unfolds^12^.

Extrapolating from Fig. 3, the maximum or complete (100%) denaturation is denoted by a DoD value of 1.0. Displayed here, thermal incubation of the S1-His protein at 75 °C for 60 min will result in the highest degree of denaturation of the S1 protein samples, represented by a DoD of ~ 0.99.

To apply the electric field of the microwave on the spike protein, we did experiments on both the S1-His sample and on the S1 protein S1-Fc. The 2, 5, and 10 min trials resulted in percent denaturation of 95, 96, and 97 of S1-Fc, respectively (Fig. 4). This is suggestive that the electric field is much more effective than temperature against Covid-19 virus. Considering within 10 min we got 97 percent denaturation to determine the efficacy against another spike protein to see if this is a general result or not, we also exposed the S1-Fc to 10 min of microwave radiation, resulting in 89 percent denaturation (Fig. 4), indicating exposure to such an external electromagnetic field significantly denatures the protein, although to slightly different degrees.

**Figure 6:**
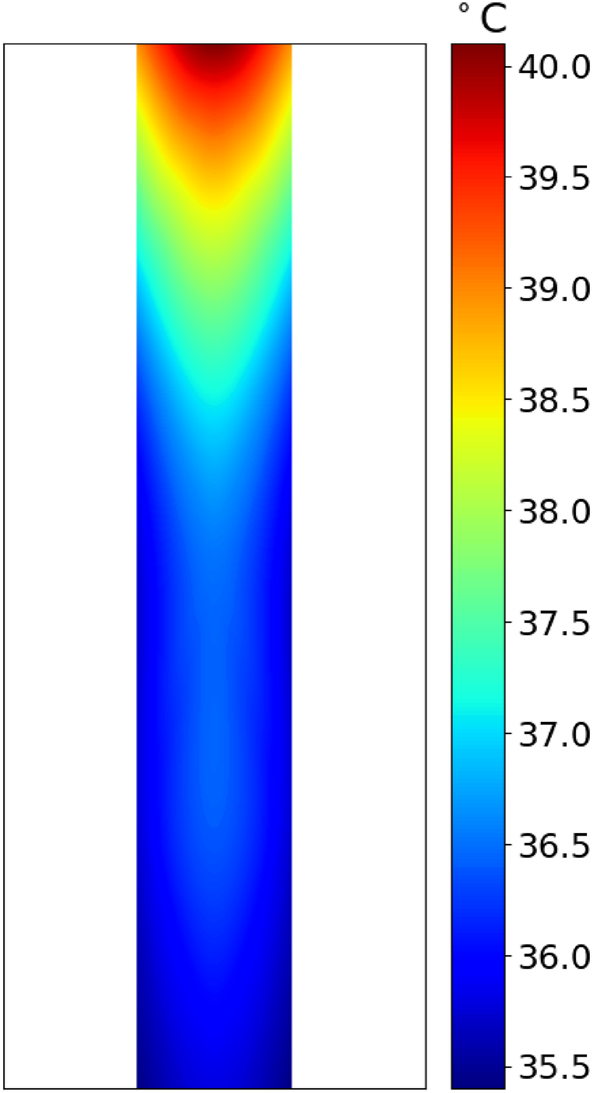
The temperature profile of the solution in the microwave reactor under microwave irradiation. The average temperature is 37 ± 1.3 °C.

Considering that the S1-His was a protein with molecular mass of 76.5 kDa and contained 681 amino acids, whereas the S1-Fc had molecular mass of 101.7 kDa and contained 908 amino acids, the electric field is more effective to denature the smaller protein. Nevertheless, the electric field of the microwave at 37 °C up to 10 min is highly effective at denaturing both, in contrast to thermal heating of the protein at 75 °C that require close to an hour. This might be due to multiple effects^17,18^. The first potential effect is the presence of hot spots in mm scale in solution due to potential temperature gradient. However, our setup (the geometry of the cell/coolant and the control over microwave pulses) and the low temperature used for our microwave experiments does rule out this effect. Figure 6 shows this by displaying the temperature profile all over the sample from our in-situ measurements using our fiber optic temperature probes.

Another potential reason is the formation of hotspots in the spike protein. At microscopic levels, hotspots with temperatures significantly higher than the bulk temperature (shown in Fig. 6) may form and dissipate quickly. Such hot spots could be originated from interactions of the electromagnetic field with the salt bridges within the protein, although other parts of the protein could also be targets of hot spots. At close distances to the salt bridge (within a few Angstrom) in the protein structure, there could be extremely high temperatures, but since we have multiple orders of magnitude of surrounding water molecules, the local increase in temperature will be negligible (less than 2 °C). Figure 6 shows that the average temperature measured in situ in solution suggests very small increase in temperature at the mm scale (mm^3^). However, such an intense microscopic hot spot in the Spike protein would inevitably cause significant damage to the Spike protein.

Another effect arises from the direct interactions of microwave electric field and the proteins. One direct interaction could be via the surface charges on the protein. The oscillating electric field of the microwave irradiation interacts with charges in different parts of the protein and exerts a force in different directions, possibly disrupting bonds and inducing denaturation within the protein structure more effectively than thermal denaturation.

Another direct interaction could be the breakdown of the directional order of the water molecules bound via hydrogen bonds to the protein and water molecules. Liquid water has a large impact on life of species and has countless biochemical properties. The hydrogen bond network of liquid water around a biomolecule is generally accepted to play a vital role on these properties. Some of the cooperative molecular motions of hydrogen bonded systems are with frequencies in the gigahertz (GHz) range that we irradiate the sample with and therefore our irradiation can strongly influence the dynamics of such intermolecular modes. The microwave radiation increases these dynamic intermolecular dynamic modes and may affect the rate and geometry of H-bond exchanges in favor of protein water H-bond breakdown and consequently the protein-water network and protein deformation^19^. Further investigation of the nature of interaction of microwave and spike proteins in contact with water is required both theoretically and experimentally.

The dashed lines are fits to bi-exponential functions explaining the time-dependence. The bi-exponential fits were the best fits globally. The exponential nature shows the process is a first order process. This suggests that the effect on proteins (and therefore on virus) is faster at higher concentration of proteins (larger viral loads). Moreover, the bi-exponential nature suggests there is a fast inactivation effect followed by a slower one. It can be also deduced that the effect of the electric field of microwaves is much more drastic and faster than that of medium temperature.

## Conclusion

The S-protein consists of two subunits, S1 and S2, previously established to participate in the binding process to the angiotensin converting enzyme 2 (ACE2) receptor^20–22^. Through the interaction of these subunits with cellular machinery, the virus is able to gain access to the cell. Heptad repeat 1 (HR1) and heptad repeat 2 (HR2) interact with the S2 subunit, establishing a six-helix bundle (6HB), guiding the S1 receptor binding domain onto the ACE2 receptor^22–24^. Subsequently, the S1 and S2 subunits are cleaved by intracellular protease, TMPRSS2, followed by endocytosis of the virus into the cell^20,22^. Upon gaining entry, viral RNA is released, and the replication process of SARS-CoV-2 is initiated^22^. Thus, it can be reasonably speculated that denaturing the associated S-protein results in prohibition of viral entry into the host cell^21,25,26^. Based on the work established here, a high degree of denaturation of the S1 protein was shown as a function of temperature and time. It was also discovered that under body-temperature (37 °C) conditions, significant denaturation can be induced by microwave irradiation anywhere from 2 to 10 min. These findings imply a potential sterilization effect for microwave approaches geared towards disinfecting surfaces contaminated by SARS-CoV-2. Additionally, should such a process be implemented in vitro, perhaps denaturation of binding domains within the S1 subunit will prevent SARS-CoV-2 infection of human cells. To that end, the possibility of disease treatment utilizing such a method is substantial.

## Methods

The SARS-CoV-2 S1-His and S1-Fc subunit recombinant proteins were purchased from Sino Biological Inc. A 5 × 10^–7^ M S1 protein solution of each was prepared in deionized water and divided into 130 µL sample batches. The UV spectrum of the natural protein was obtained using the Mettler Toledo UV5Nano UV–visible spectrophotometer, equipped with both microliter measurement capacity and 100 µL microcuvettes. DI water was used as the blank. For thermal denaturation of the S1-His protein, three incubation temperatures were chosen: 50, 60 and 75 °C. For each temperature, the sample was transferred to a sealed Eppendorf tube and was heat-treated in a water bath at the chosen temperature for six incubation times: 5, 10, 20, 30, 40 and 60 min, using a new sample for each temperature. Following each heat treatment, the sample was subsequently removed and left to sit for 2 min to reach equilibrium with room temperature. UV–visible analysis was then performed on the heat-treated sample to measure absorbance, and the procedure was repeated for each temperature sample group. The raw data from the UV spectrophotometer was analyzed using a Python program, developed in house.

**Figure 7:**
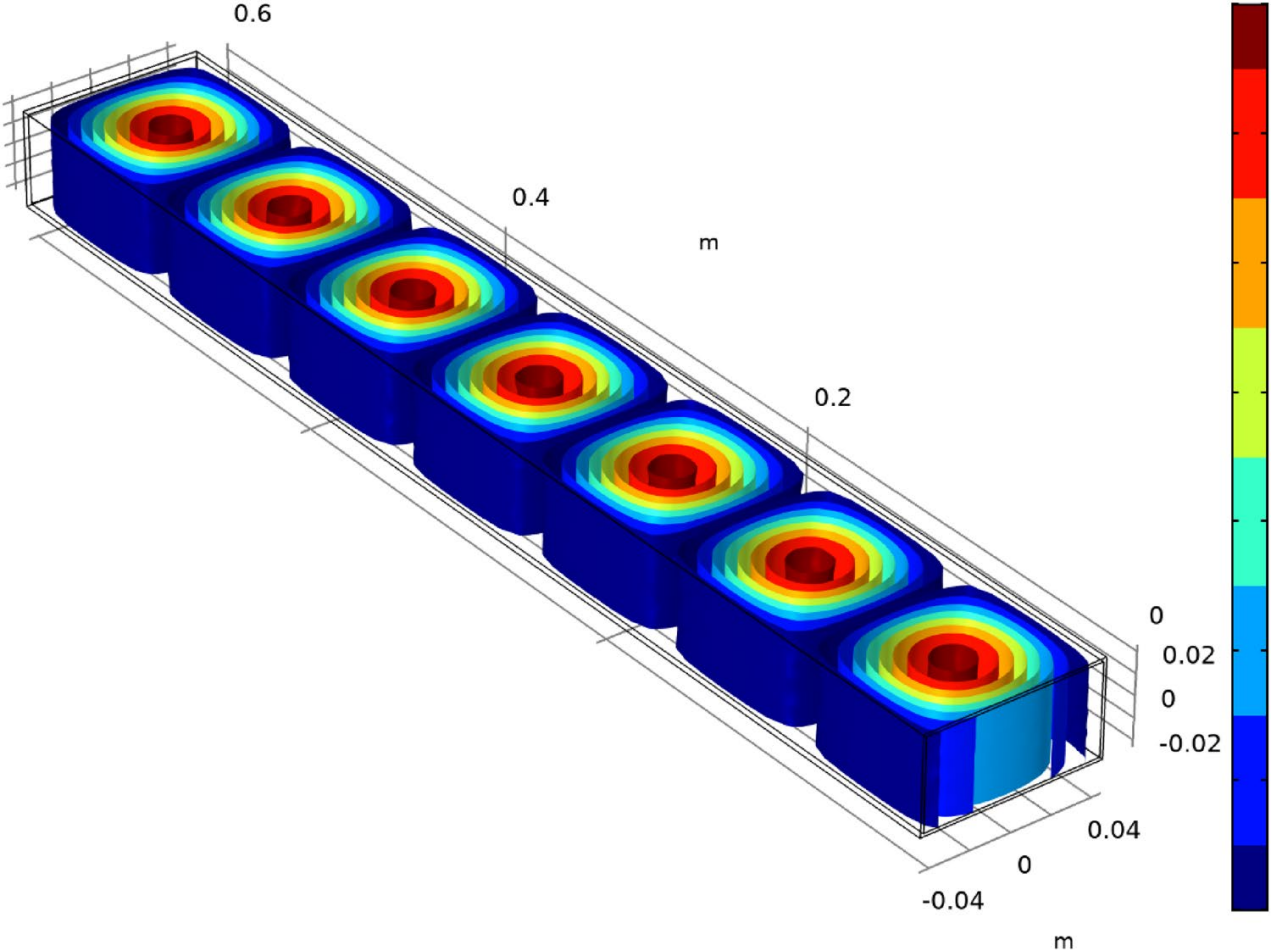
The simulated electric field propagation of 2.45 GHz microwave inside our waveguide. The color bar scale is arbitrary and is a measure of the electric field in V/m.

An inventive microwave system that has been previously designed and developed in house was used for this study (Fig. 1)^27^. The system is capable of delivering microwave energy at 2.45 GHz with tunable power and pulses, utilizing a tailored sample chamber and cooling system to effectively cool a sample (Fig. 1). The microwave generator was a water-cooled magnetron with maximum output of 1000 W at 2.45 GHz, equipped with isolator and 3-stub tuner. The output microwaves were guided to the sample through a waveguide which can only allow one TEM mode at this frequency (TE10). Figure 7 shows the simulated electric field inside the waveguide forming standing waves. The sample chamber is positioned at the end of the waveguide. The exact position of the sample chamber is where there is maximum electric field through measurements. The very small dimensions of the sample tube (~ 3 mm diameter and ~ 20 mm length) compared to the microwave wavelength (~ 12 cm), ensures that the microwave fields are uniform throughout the sample. The stub tuners were adjusted in such way to have the maximum impedance matching and maximum microwave absorption in the sample. The impedance adjustment was done by measuring both the incident and reflected microwave powers with diodes inside the waveguide. More details (and the data) about the reliability of the system can be found in the supplementary information S1. The reflected power was less than 5% of the total incident power. Inside the waveguide was filled with standard dry air for all the experiments. A software developed by the authors was used to automatically control the microwave pulse in conjunction with the control of the temperature of the coolant fluid and maintain the temperature of samples at any desired level by reading the in situ temperature (using fiber optics sensors). The optical thermometer system (fiber optic) made by OSENSA had an accuracy of ± 0.1 °C and response time of 250 ms.

Using similar spectroscopic process and protein concentrations as above, the UV spectrum of the natural protein sample was obtained. The sample was then transferred to the sample chamber inside the microwave setup waveguide. As a control, the natural protein solutions (S1-His and S1-Fc) were transferred to the microwave sample chamber inside the waveguide without turning on the microwave source, therefore, no denaturation effect was observed. To investigate the degree of denaturation (DoD) of the proteins in this study, a fast, reproducible, and non-destructive optical UV spectroscopy data analysis technique was used, as previously developed by the authors^12^.

## Supplementary Information

Supplementary Information.

## Data Availability

The datasets and the computer codes that were used to generate and/or analyze the results during the current study are available from the corresponding author on reasonable request.
