# Supplementary material for: Denaturation of the SARS-CoV-2 spike protein under non-thermal microwave radiation
Source: Sci Rep. 2021 Dec 3;11:23373. doi: 10.1038/s41598-021-02753-7 (PMC8642515; doi:10.1038/s41598-021-02753-7)
Supplement: Supplementary file 1 — Supplementary Information. [file 41598_2021_2753_MOESM1_ESM.pdf]

Supplementary Information

Figure S1 shows the incident microwave power (yellow) and the backward/reflected microwave power (blue) measured with two diodes placed on the waveguide connected to an oscilloscope. The stub tuners were adjusted in such a way to maximize the microwave absorption in the sample. More than 95% of the total microwave power was absorbed. This also shows the stability of the microwave radiation with minimal fluctuations. No measurements were done with larger fluctuations in the power.

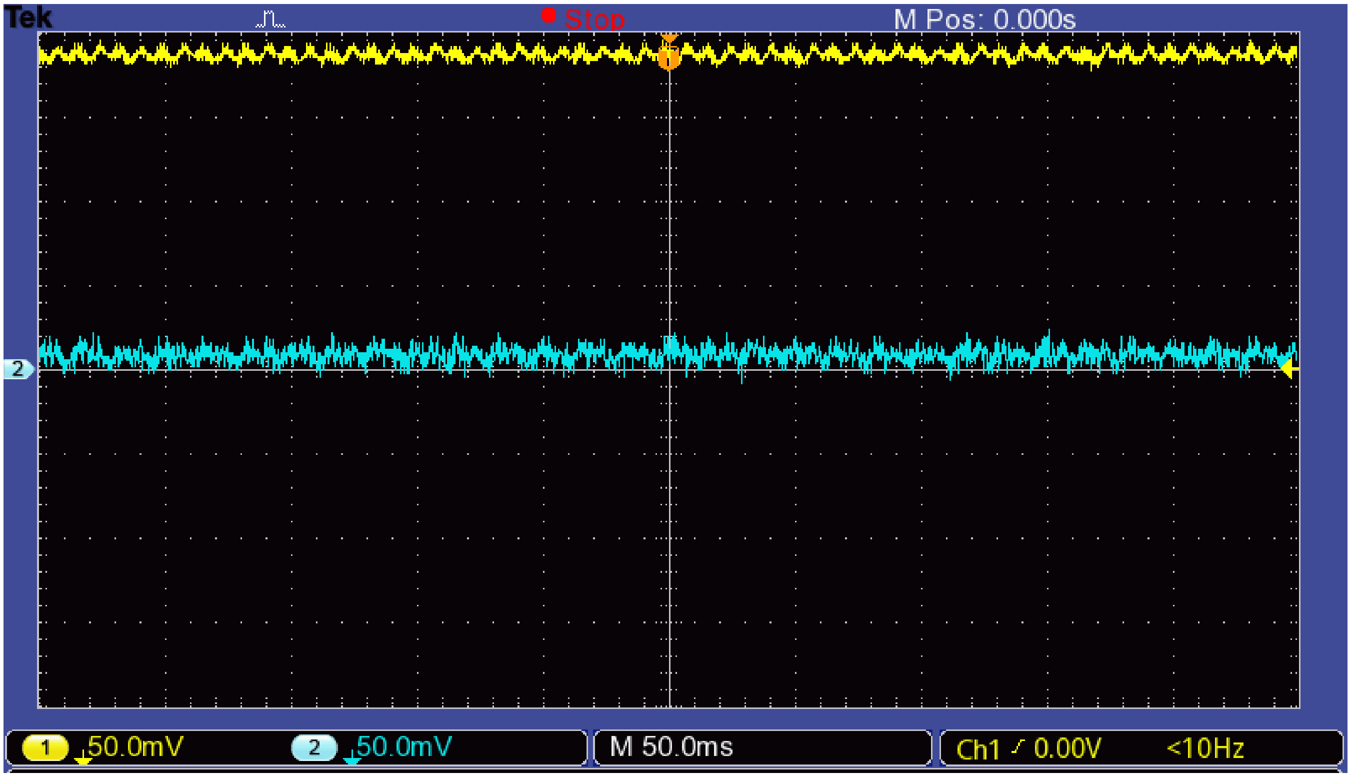

Figure S1. the incident microwave power (yellow) and the backward/reflected microwave power (blue).
